# Orai1 Expression and Vascular Function in Kidney Donors Determine Graft Outcomes at Short/Mid-Term

**DOI:** 10.3390/cells14131005

**Published:** 2025-07-01

**Authors:** Esther García-Rojo, Javier Angulo, Mariam El Assar, Rocío Santos-Pérez de la Blanca, Borja García-Gómez, José Medina-Polo, Alejandro Sevilleja-Ortiz, Leocadio Rodríguez-Mañas, Argentina Fernández, Eduardo Gutiérrez-Martínez, Enrique Morales-Ruiz, Alfredo Rodríguez-Antolín, Javier Romero-Otero

**Affiliations:** 1 Department of Urology, Hospital Universitario HM Sanchinarro, HM Hospitales, 28050 Madrid, Spain; esthergrojo@hotmail.com (E.G.-R.); jromerootero@hotmail.com (J.R.-O.); 2 Department of Histology-Research, Unidad de Investigación Traslacional en Cardiología (IRYCIS-UFV), Hospital Universitario Ramón y Cajal, 28034 Madrid, Spain; alex.sevilleja9335@gmail.com (A.S.-O.); argentina.fernandez@salud.madrid.org (A.F.); 3 Centro de Investigación Biomédica en Red sobre Fragilidad y Envejecimiento Saludable (CIBERFES), Instituto de Salud Carlos III, 28029 Madrid, Spain; melassar@iisgetafe.com (M.E.A.); leocadio.rodriguez@salud.madrid.org (L.R.-M.); 4 Instituto de Investigación IdiPaz, 28029 Madrid, Spain; 5 Fundación para la Investigación Biomédica, Hospital Universitario de Getafe, 28905 Getafe, Spain; 6 Department of Urology, Hospital Universitario 12 de Octubre, Instituto de Investigación Sanitaria Hospital 12 de Octubre (imas12), 28041 Madrid, Spain; rociosantosperez@hotmail.com (R.S.-P.d.l.B.); gendine@hotmail.com (B.G.-G.); josemedinapolo@movistar.es (J.M.-P.); arantolin@yahoo.es (A.R.-A.); 7 Department of Geriatrics, Hospital Universitario de Getafe, 28905 Getafe, Spain; 8 Department of Nephrology, Hospital Universitario 12 de Octubre, Instituto de Investigación Sanitaria Hospital 12 de Octubre (imas12), 28041 Madrid, Spain; eduardogutmat90@gmail.com (E.G.-M.); emorales@senefro.org (E.M.-R.)

**Keywords:** kidney transplantation, renal function, Orai calcium channels, endothelial vasodilation, adrenergic vasoconstriction, human mesenteric artery, human aorta, human corpus cavernosum, serum creatinine, glomerular filtration rate

## Abstract

We aimed to determine the influence of donors’ vascular function on renal function in recipients and to evaluate the role of Orai1 calcium channels as a potential marker. A prospective collaborative multicenter study was designed. Blood, aorta (HA), mesenteric arteries (HMAs) and corpus cavernosum (HCC) specimens were obtained from organ donors at the kidney procurement procedure (n = 60). Evolution (up to 2 years) of renal function measured as serum creatinine (SCr) and glomerular filtration rate (GFR) was evaluated in respective recipients (n = 64). Vascular responses were determined in HA, HMA and HCC from donors. Tumor necrosis factor-α, asymmetric dimethyl arginine and Orai1 were measured in plasma. Orai1 protein expression was also evaluated in each donor’s aorta. Endothelium-dependent vasodilation (HMA, HCC) and adrenergic contraction (HA) in donors determined renal function in recipients, 12 months post-transplantation. Donors in the best quartile of vascular function predicted lower SCr and higher GFR in kidney recipients for 12/24 months. Plasma Orai1 in donors was negatively correlated with vascular function and predicted renal function at 3–6 months post-transplantation. Donor Orai1 vascular content was associated with reduced vascular function and with poorer recipient renal function for 1-year post-transplantation. Systemic vascular function of kidney donors determines recipients’ renal function at short/mid-term. Donors’ vascular function and recipients’ renal function are negatively associated with donors’ Orai1 vascular expression, being a potential biomarker of renal outcomes.

## 1. Introduction

Kidney disease is a highly prevalent condition and represents a growing global problem due to population dynamics and the rise of environmental and social threats [1]. Specifically, chronic kidney disease (CKD) resulted in 1.2 million deaths in 2017 [2]. The best option in terms of survival and quality of life (QoL) for end-stage CKD patients is kidney transplantation, using both grafts from deceased and living donors [3,4,5]. Due to the high demand and limited supply of organs [6], it is of utmost importance to identify the conditions of kidney donors, which determine the clinical success of transplantation. However, there is still a lack of fundamental and longitudinal research on accurate predictors or biomarkers of early and late graft dysfunction to prolong graft survival [7].

Kidney transplantation success can be compromised by multiple factors [8]. Moreover, renal function is highly dependent on adequate vascular function, and this is especially relevant when considering the increase in the use of marginal kidney donors [9]. Vascular territories exhibit significant heterogeneity, especially at the functional level [10], and the impact of cardiovascular risk factors (CVRFs) differs across each vascular bed [11]. For instance, erectile dysfunction (ED), which is highly prevalent in men with CKD, especially among those on dialysis [12], is considered an early marker for cardiovascular disease (CVD) and represents a determinant factor in the development of cardiovascular manifestation [4,5]. To our knowledge, there are no studies analyzing the relationship between the donor’s vascular function and clinical evolution of the grafts.

Several markers of vascular function have been proposed. These include the endogenous inhibitor of nitric oxide (NO) synthase, asymmetric dimethyl-arginine (ADMA), considered as a marker of endothelial dysfunction and CVD risk [13,14,15], and inflammatory cytokines such as tumor necrosis factor-α (TNF-α), which is associated with vascular pathophysiology [16,17]. Recently, the upregulation of the Orai calcium channel-forming subunits involved in the store-operated calcium entry (SOCE) has been proposed as a mechanism contributing to vascular impairments related to aging and disease [18,19,20,21]. The role of Orai1 in renal pathophysiology has been suggested but not elucidated [22]. It has been proposed to participate in mesangial cell proliferation [23] and albumin endocytosis [24] as well as to mediate fibroblast growth factor-23 increase [25] and to be upregulated in kidneys from mice with induced nephropathy [26] and from diabetic rats [27].

The aim of this study was to evaluate the influence of donor’s vascular function across different vascular beds on the evolution of renal function in kidney recipients. The potential role of Orai1 as a marker of both vascular function in donors and renal outcome in kidney recipients was also explored.

## 2. Materials and Methods

### 2.1. Study Subjects

Adult organ donors providing one or two kidneys for implantation to one or two recipients at the Renal Transplant Unit of the Hospital Universitario 12 de Octubre in Madrid, Spain, were included. Donors after either circulatory or brain death were included. Donors with infectious diseases were excluded. Only adult recipients corresponding to the included donors were selected. Among them, recipients with less than 6 months follow-up were excluded. The study was conducted in accordance with the principles outlined in the Declaration of Helsinki. Donors’ relatives and recipients provided written informed consent before being included. Protocols and consent forms were approved by the Ethics Committees of the three Hospitals involved (Ethics Approval procedures: 16/045 (12 de Octubre University Hospital, approval 25 February 2016), 363-15 (Ramón y Cajal University Hospital, approval 3 March 2016), and A06/15 (Getafe University Hospital, approval 30 April 2015)).

### 2.2. Clinical Variables and Follow-Up

Different clinical variables, type of death (brain death or asystole and type) and comorbidities such as diabetes mellitus, hypertension, dyslipidemia or CVD were recorded from donors. Some blood test parameters were noted before organ procurement (serum creatinine (SCr), glomerular filtration rate (GFR) (calculated by using the Chronic kidney Disease Epidemiology collaboration (CKD-EPI) formula) [28] and proteinuria), as well as the presence of hypotension or the need to use vasoactive drugs in the hours preceding death. Donor-specific antibodies for the different human leukocyte antigens (HLAs) were evaluated and the number of HLA incompatibilities with the recipient were determined.

Regarding the recipients, general variables were collected together with the cause of CKD, type and time on dialysis, data related to the transplantation such as ischemia times, type of immunosuppression and the number of HLA incompatibilities. The graft recipients underwent the standard follow-up procedure at the Renal Transplant Unit, with no deviation from routine clinical practice. Different analytical parameters including SCr, GFR, proteinuria, albumin, uric acid, calcium or phosphorus were collected during evolution, obtaining data at 3, 6, 12 and 24 months after transplantation.

### 2.3. Donor Tissues

Blood samples and vessel specimens (aorta, omentum and corpus cavernosum in male patients) were obtained from organ donors at the time of organ procurement. Tissues were maintained at 4–6 °C in sterilized M-400 solution (composition per 100 mL: mannitol, 4.19 g; KH_2_PO_4_, 0.205 g; K_2_HPO_4_, 0.97 g; KCl, 0.112 g; NaHCO_3_, 0.084 g; pH 7.4) and transported in refrigerated conditions to laboratories in collaborating hospitals. Time from extraction to processing ranged between 16 and 24 h [20,29].

### 2.4. Human Mesenteric Arteries (HMAs)

Mesenteric small vessels (lumen 150–500 µm), were dissected from omentum specimens, and arterial ring segments (1.5–2 mm) were subsequently mounted on wire myographs (Danish MyoTechnology, Aarhus, Denmark) for isometric tension recordings [21,29]. The vessels were allowed to equilibrate for 30 min in Krebs–Henseleit solution (KHS), which has the following composition (mM): NaCl 119, KCl 4.6, CaCl_2_ 1.5, MgSO_4_ 1.2, NaHCO_3_ 24.9, glucose 11, KH_2_PO_4_ 1.2, EDTA 0.027; at 37 °C, the vessels were continuously bubbled with 95% O_2_/5% CO_2_ mixture to maintain a pH of 7.4. The arteries were then set to 90% of the determined internal circumference under a transmural pressure of 100 mmHg (L_100_). Preparations were then exposed to high K^+^ concentration (125 mM), and the contractile response was measured. For relaxation experiments, arterial segments were precontracted with the thromboxane analogue, U46619 (10–100 nM, 80% of K^+^-induced contraction). When a stable plateau was reached, endothelium-dependent vasodilatory responses were evaluated by cumulative addition of bradykinin (BK; 10 nM–3 µM).

### 2.5. Human Aorta (HA)

Human aortic segments were cut into transversal strips of about 7 mm in length and 2 mm in width and immersed in 8 mL organ chambers containing KHS bubbled with 95% O_2_/5% CO_2_ mixture and maintained at 37 °C as previously described [20,29]. Strips were allowed to equilibrate for 90 min at 1.5 g of tension. Strips were exposed to high K^+^ concentration (125 mM) and the contractile response was measured. Contraction responses were evaluated by cumulative additions of norepinephrine (NE; 1 nM–100 µM).

### 2.6. Human Corpus Cavernosum (HCC)

Strips of corpus cavernosum tissue obtained from male donors were immersed in 8 mL organ chambers as described for aortic strips. Each tissue strip was incrementally stretched to optimal isometric tension, as previously described [18,29]. Strips were exposed to high K^+^ concentration (125 mM) and the contractile response was measured. For relaxation experiments, cavernosal strips were precontracted with phenylephrine (1–3 µM, 80% of K^+^-induced contraction). When a stable plateau was reached, endothelium-dependent relaxation responses were evaluated by cumulative addition of acetylcholine (ACh, 1 nM–10 µM).

### 2.7. Biomarker Assessment

When possible, blood from donors and recipients was collected in sodium-EDTA tubes and centrifuged at 4 °C to obtain plasma. Plasma was frozen in aliquots until determination of biomarkers. ADMA, TNF-α and Orai1 were determined in plasma by ELISA using commercially available kits (CloudClone Corp., Katy, TX, USA, R&D Systems, Minneapolis, MN, USA, and MyBiosource, San Diego, CA, USA, respectively) and following manufacturers’ instructions.

### 2.8. Immunofluorescence Assay

Freshly isolated aorta specimens were immersed in increasing percentages of saccharose (10–30% *w*/*v*), embedded in OCT and kept at −80 °C until the immunofluorescence assays were carried out, as described previously [30]. Then, 6 μm sections were incubated at 4 °C overnight with rabbit antibody against Orai1 (1:200 dilution; Novus Biologicals, Littleton, CO, USA). After washout in phosphate buffered saline plus 0.3% Triton X-100, the sections were incubated with a secondary Alexa Fluor 488-conjugated goat anti-rabbit antibody (dilution 1:250; Life Technologies, Alcobendas, Spain) and with diamidino-2-phenylindole (DAPI; Life Technologies) to counterstain the nuclei. The sections were mounted and viewed using fluorescence microscopy. Controls without primary antibodies did not show unspecific reactivity.

### 2.9. Western Blot

Western blot analyses were carried out as previously described [20]. Aortic tissue samples derived from organ donors were flash frozen in liquid nitrogen and kept at −80 °C until the proteins were extracted. For obtaining total protein extracts, aortic tissue was homogenized in a T-PER lysis buffer (Pierce Biotechnology, Inc., Rockford, IL, USA) with Protease Inhibitor Cocktail (Roche Diagnostics, Indianapolis, IN, USA). Proteins (15 μg) were separated via SDS-PAGE on a 10% polyacrylamide gel, transferred to PVDF membranes and blocked with EveryBlot blocking buffer (Bio-Rad, Hercules, CA, USA). The membranes were incubated overnight at 4 °C with a specific monoclonal mouse antibody against Orai1 (ThermoFisher Scientific, Waltham, MA, USA, cat.# MA5-15776, dilution 1:500) and a monoclonal mouse antibody against β-actin (Novus, cat.# NB600-501, dilution 1:5000), which was used as the loading control. Consequently, the membranes were incubated with goat anti-mouse antibody (1:5000 dilution; Novus, cat.# NBP2-30347H). The blots were visualized with the ECL detection system (ThermoFisher Scientific). The results were quantified by densitometry, using QuantityOne/Chemi-Doc 6.0 Software (Bio-Rad, Barcelona, Spain).

### 2.10. Data Analysis

The categorical variables were expressed in absolute numbers and percentages. The quantitative variables were presented as means ± standard deviations (SD). Vascular function was expressed as the –log of the molar concentration of BK or ACh for obtaining 50% relaxation (pEC_50_) or as the maximum contraction to NE (E_max_). Parameters of donors’ vascular function in each tissue were related to parameters of renal function during recipients’ evolution using Pearson correlation coefficient. For evaluating potential interferences, a mixed model with regression analyses was performed, using the evolution variables as fixed effects and the donor variables as covariates. Grouped data were compared via non-parametric Mann–Whitney U-test. The statistical analysis was performed with the statistical software SAS^®^ version 9.4 (SAS Institute, Cary, NC, USA) and the GraphPad Prism software 8.0 (San Diego, CA, USA). A *p*-value < 0.05 was considered statistically significant.

## 3. Results

### 3.1. Baseline Characteristics of Study Subjects

Samples corresponding to 60 donors (15 women and 45 men) were obtained. The main clinical characteristics are displayed in Table 1. Organs and tissues were collected from 41 subjects at brain death and from 19 subjects after circulatory death (4 patients, 21.0% Type I, 6 patients, 31.6% type II and 9 patients, 47.4% type III asystole). We found that 31% presented hypotension and 39.2% needed the use of vasoactive drugs in the hours preceding death. About one third of donors were classified as Expanded Criteria Donors (ECDs) [31]. Blood tests before organ procurement displayed 1.02 ± 0.54 mg/dL of SCr, 79.44 ± 22.21 mL/min of estimated GFR and 0.30 ± 0.37 mg/dL of proteinuria.

Baseline clinical characteristics of the 64 recipients (21 women and 43 men) are summarized in Table 1. Patients were 26.2 ± 19.3 months on dialysis substitutive treatment before receiving the graft. A total of 51 patients (80.9%) received their first kidney graft, 9 (14.3%) received a second, 2 (3.2%) a third one and 1 (1.6%) a fourth graft. We found 4.19 ± 1.26 HLA incompatibilities between donors and recipients. Cold ischemia time was 938.15 ± 364.45 min.

### 3.2. Donors’ Vascular Function and Evolution of Recipients’ Renal Function

**Figure 1:**
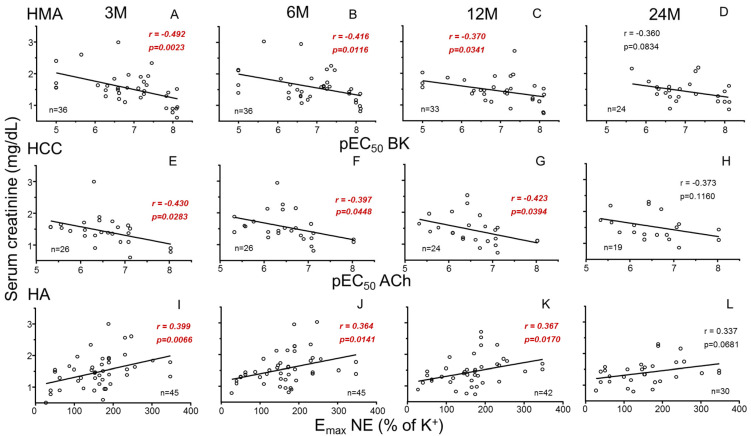
Correlations between parameters of systemic vascular function of donors and evolution of recipients’ serum creatinine. Graphs show correlations of endothelial vasodilation (X-axis) in human mesenteric arteries (HMAs) determined as −log molar of concentration required to obtain 50% relaxation (pEC_50_) for bradykinin (BK) (**A**–**D**), endothelial relaxation in human corpus cavernosum (HCC) determined as pEC_50_ for acetylcholine (ACh) (**E**–**H**), and adrenergic contraction in human aorta (HA) determined as the maximum response (E_max_) to norepinephrine (NE) expressed as the percentage of K^+^-induced contraction (**I**–**L**) from kidney donors with serum creatinine concentrations (Y-axis, mg/dL) in respective kidney recipients at 3 (3 M), 6 (6 M), 12 (12 M) and 24 months (24 M) after transplantation. Correlation coefficients and *p*-values are indicated for each correlation. Significant associations are highlighted by *p*-values in red. n indicates the number of determinations.

**Figure 2:**
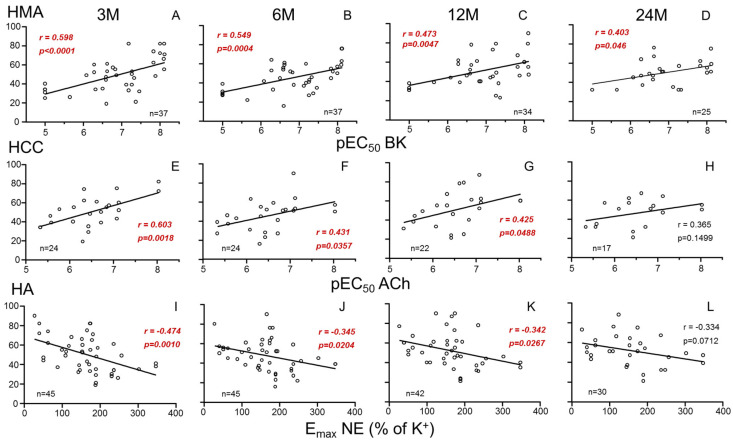
Correlations between parameters of systemic vascular function of donors and evolution of recipients’ glomerular filtration rate (GFR). Graphs show correlations of endothelial vasodilation (X-axis) in human mesenteric arteries (HMAs) determined as −log molar of concentration required to obtain 50% relaxation (pEC_50_) for bradykinin (BK) (**A**–**D**), endothelial relaxation in human corpus cavernosum (HCC) determined as pEC_50_ for acetylcholine (ACh) (**E**–**H**), and adrenergic contraction in human aorta (HA) determined as the maximum response (E_max_) to norepinephrine (NE) expressed as the percentage of K^+^-induced contraction (**I**–**L**) from kidney donors with glomerular filtration rate (Y-axis, mL/min/1.73 m^2^), in respective kidney recipients at 3 (3 M), 6 (6 M), 12 (12 M) and 24 months (24 M) after transplantation. Correlation coefficients and *p*-values are indicated for each correlation. Significant associations are highlighted by *p*-values in red. n indicates the number of determinations.

Endothelium-dependent vasodilation (pEC_50_ for BK) in mesenteric vasculature (HMA) of donors was significantly and negatively associated with recipients’ SCr and positively with recipients’ GFR at 3 months after transplantation. This association persisted at 6 and 12 months but lost significance at 24 months (Figure 1A–D and Figure 2A–D for SCr and GFR, respectively).

Recipients’ SCr was also negatively associated with endothelium-dependent relaxation (pEC_50_ for ACh) in the penile corpus cavernosum (HCC) from male donors at 3, 6 and 12 months (Figure 1E–H). Consistently, endothelial response in HCC was positively related to GFR (Figure 2E–H). In contrast, adrenergic vasoconstriction (E_max_ to NE) in donors’ aortae (HA) was positively correlated with recipients SCr (Figure 1I–L) and negatively correlated with recipients’ GFR (Figure 2I–L) at 3, 6 and 12 months after transplantation. No statistically significant correlations (*p* > 0.05) were found between vascular function parameters and other analytical values of clinical evolution (proteinuria, albumin, uric acid, calcium, phosphorus).

Furthermore, adjustment models were employed to account for potential confounding variables influencing the relationship between donor vascular function and renal function in kidney recipients. Despite significant influences of variables such as age, type of death or donor creatinine prior to extraction, associations remained significant after adjusting for those variables (Table 2). This confirms the specific determinant role of systemic vascular function of kidney donors on renal outcomes in recipients. The time of evolution itself did not act as a confounding variable for these relationships.

On the other hand, to ascertain the potential influence of cold ischemia time on kidney function, we evaluated the relationship of cold ischemia time in transplantation procedure with SCr and GFR at different periods of follow-up. There were no significant associations of cold ischemia time in minutes with SCr and GFR at any post-transplantation time evaluated (*p* = 0.4903, *p* = 0.6171, *p* = 0.7356 and *p* = 0.5210 for SCr at 3, 6, 12 and 24 months, respectively, and *p* = 0.9233, *p* = 0.8045, *p* = 0.6169 and *p* = 0.2934 for GFR at 3, 6, 12 and 24 months, respectively).

**Figure 3:**
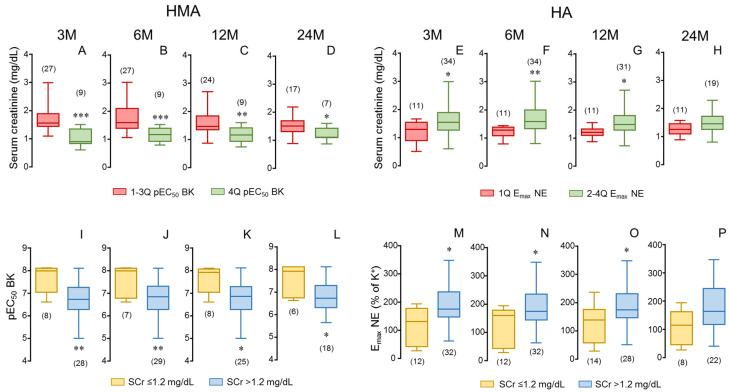
Stratification of endothelial vasodilation and adrenergic contractility in donors as well as stratification of serum creatinine (SCr) in kidney recipients confirms the determinant role of donor vascular function in recipient renal function. Upper panels show SCr concentrations of kidney recipients at specific evolution times (3 (3 M), 6 (6 M), 12 (12 M) and 24 months (24 M) after transplantation) corresponding to donors displaying the highest vasodilatory response in human mesenteric arteries (HMAs, fourth quartile of pEC_50_ for bradykinin; 4 Q) compared to recipients corresponding to the other donors (in first to third quartiles of pEC_50_ for BK; 1–3 Q) (**A**–**D**) as well as to donors displaying the lowest contractility in aorta (HA, first quartile of E_max_ to norepinephrine (NE); 1 Q) compared to recipients corresponding to the other donors (in second to fourth quartiles of E_max_ to NE; 2–4 Q) (**E**–**H**). Lower panels show endothelial vasodilation of HMAs determined as pEC_50_ for BK (**I**–**L**) and adrenergic contraction in HA determined as the E_max_ to NE (**M**–**P**) of donors corresponding to kidney recipients divided into those displaying SCr concentrations equal or below 1.2 mg/dL (≤1.2) and those displaying higher creatinine levels (>1.2) at specific evolution times. Data are summarized in box-and-whiskers plots with complete range. The number of recipients (**upper panels**) and donors (**lower panels**) are indicated in parentheses. * *p* < 0.05, ** *p* < 0.01, *** *p* < 0.001 by Mann–Whitney U-test.

The determinant role of donor’s vascular function on renal outcomes in transplantation was further confirmed when donors were stratified by their vascular function. Considering endothelial vasodilation in HMAs, donors in the highest quartile of pEC_50_ for BK were related to kidney recipients displaying significantly lower levels of SCr when compared to all the other recipients in every time determination until 24 months (Figure 3A–D). An analogous pattern was observed when considering adrenergic contractility in aortae. Donors in the lowest quartile of E_max_ of NE-induced contraction in aorta were associated with kidney recipients having lower SCr concentrations, although the significance was lost at 24 months post-transplantation (Figure 3E–H). When recipient renal function was determined by GFR values, a similar pattern was obtained (Supplementary Figure S1). Further analyses reinforced the association of donor vascular function with renal function outcome in kidney recipients. When analyzing vascular function in recipients displaying normal SCr values (equal or below 1.2 mg/dL), it was revealed that they had received kidneys from donors with significantly better endothelial function, evidenced by higher values of pEC_50_ for BK in HMAs (Figure 3I–L), while these donors also showed significantly lower contractility to NE in the aorta (Figure 3M–P). This pattern was observed during the 24-month evolution period for endothelial vasodilation in HMAs (Figure 3I–L) while significance for adrenergic contraction of HA was lost at the longest evolution period (Figure 3M–P).

### 3.3. Plasma Biomarkers Related to Donor Vascular Function and Recipient Renal Function

**Figure 4:**
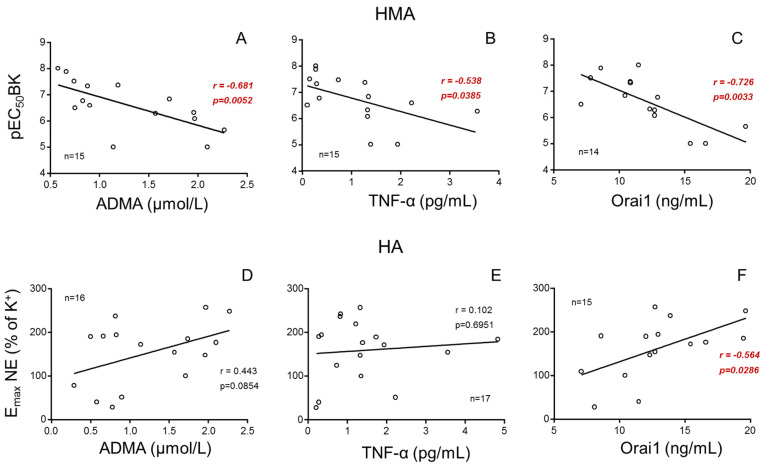
Plasmatic concentrations of ADMA, TNF-α and Orai1 are associated with vascular responses in kidney donors. Influence of plasma concentrations of asymmetric dimethyl arginine (ADMA), tumor necrosis factor-α (TNF-α) and Orai1 on vasodilatory response in human mesenteric arteries (HMAs, (**A**–**C**)) determined as the pEC_50_ for bradykinin (BK) and on adrenergic contractility in human aorta (HA, (**D**–**F**)) determined as the E_max_ to norepinephrine (NE). Correlation coefficients and *p*-values are indicated for each correlation. Significant associations are highlighted by *p*-values in red. n indicates the number of determinations.

When plasma sample was available, its concentrations of the inflammatory cytokine, TNF-α, the endogenous NO-synthase inhibitor, ADMA, and the calcium channel, Orai1, were determined. In donors, endothelial vasodilation of HMAs significantly and negatively correlated to plasma concentrations of ADMA, TNF-α and Orai1 (Figure 4A–C) while contractile responses in HA only significantly and positively correlated with Orai1 in plasma (Figure 4D–F).

**Figure 5:**
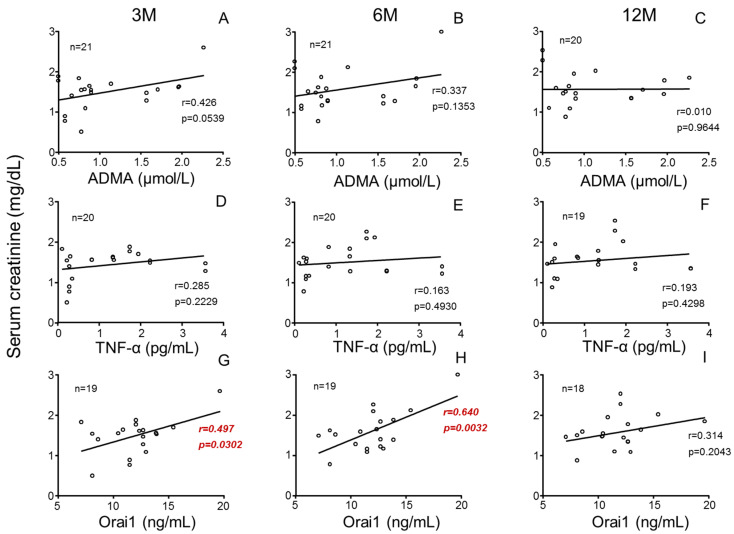
Donors’ plasmatic concentrations of Orai1 but not those of ADMA or TNF-α are positively associated with serum creatinine concentrations in kidney recipients. Influence of donors’ plasma concentrations of asymmetric dimethyl arginine (ADMA) (**A**–**C**), tumor necrosis factor-α (TNF-α) (**D**–**F**) and Orai1 (**G**–**I**) on serum creatinine concentrations in kidney recipients at specific evolution times (3 (3 M), 6 (6 M) and 12 months (12 M) after transplantation). Correlation coefficients and *p*-values are indicated for each correlation. Significant associations are highlighted by *p*-values in red. n indicates the number of determinations.

Furthermore, while plasma levels of ADMA (Figure 5A–C) and TNF-α (Figure 5D–F) failed to be associated with recipient renal function, plasma Orai1 concentrations were significantly associated with increasing recipient SCr concentrations (Figure 5G,H) as well as with decreasing GFR (Supplementary Figure S2) at 3 and 6 months post-transplantation. This relationship was not significant at 12 months after transplantation (Figure 5I). In contrast to donors, the concentrations of these biomarkers in plasma from kidney recipients did not display any significant association with their renal function after transplantation (Supplementary Figure S3).

### 3.4. Renal Function in Kidney Recipient Is Predicted by Vascular Expression of Orai1 in Donor

**Figure 6:**
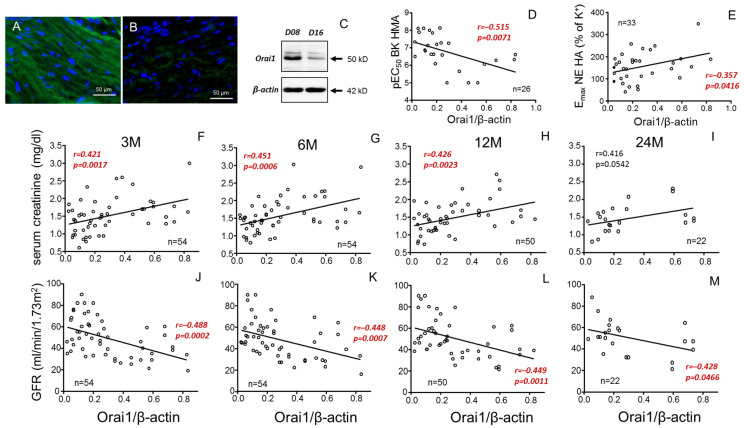
Negative correlation of donor aortic Orai1 expression with vasodilation of mesenteric arteries and recipient renal function. Panels (**A**,**B**) show immunofluorescence detection of Orai1 in smooth muscle of aortic tissues from two different donors displaying high ((**A**), D08) and low ((**B**), D16) immunostaining which is confirmed by immunoblot (**C**). Panels (**D**,**E**) show correlations of aortic expression of Orai1 expressed as Orai1/β-actin immunoblot band intensities ratio with endothelial vasodilation expressed as pEC_50_ for bradykinin (BK) in mesenteric arteries (HMAs) (**D**) and aortic vasoconstriction expressed as E_max_ for norepinephrine (NE) (**E**) from the same donors. Other panels show correlations of aortic expression of Orai1 in donors with serum creatinine concentrations (**F**–**I**) and glomerular filtration rate (GFR) (**J**–**M**) in respective kidney receptors at specific evolution times (3 (3 M), 6 (6 M), 12 (12 M) and 24 months (24 M) after transplantation). Correlation coefficients and *p*-values are indicated for each correlation. Significant associations are highlighted by *p*-values in red. n indicates the number of determinations. Complete annotated images of immunoblots for Orai1 and β-actin are provided in Supplementary Figure S4.

Considering the role of Orai1 concentrations in donor plasma for predicting vascular responses in donors and renal function in kidney recipients, the expression of Orai1 at the vascular level was evaluated. Immunofluorescence detection of Orai1 revealed the expression of these channels in aortic smooth muscle from kidney donors. However, Orai1 expression levels varied from one specimen to another (Figure 6A,B), as also evidenced by immunoblot assays (Figure 6C). The individual Orai1/β-actin ratio in aortae from donors significantly and negatively correlated with endothelial vasodilation of HMAs from the same subjects, expressed as pEC_50_ for BK (Figure 6D). On the other hand, Orai1 expression significantly and positively correlated with vasoconstriction of aortae (Figure 6E). Furthermore, aortic expression of Orai1 correlated with SC levels (positively) (Figure 6F–I) and GFR (negatively) (Figure 6J–M) in kidney recipients. This association remained significant for SCr concentrations up to 12 months after transplantation, while for GFR, even at 24 months post-transplantation, significance was still present (Figure 6M).

**Figure 7:**
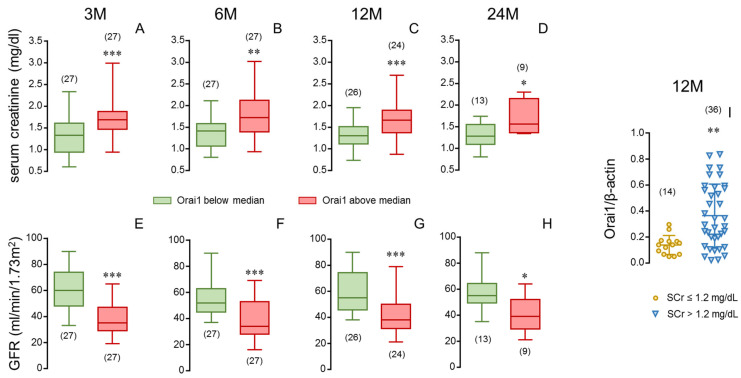
Stratification of aortic expression of Orai1 in donors and stratification of serum creatinine in kidney recipients confirm the determinant role of donor vascular Orai1 expression in recipient renal function. Serum creatinine concentrations (SCr, (**A**–**D**)) and glomerular filtration rates (GFR) (**E**–**H**) of kidney recipients at specific evolution times (3 (3 M), 6 (6 M), 12 (12 M) and 24 months (24 M) after transplantation) corresponding to donors displaying high (above median) or low (below median) aortic expression of Orai1 determined as Orai1/β-actin immunoblot band intensities ratio. Data are summarized in box-and-whiskers plots with complete range. Panel (**I**) shows aortic expression of Orai1 in donors corresponding to kidney recipients displaying SCr concentrations equal or below 1.2 mg/dL (≤1.2) and those displaying higher creatinine levels (>1.2) at 12 months after transplantation. Data are expressed as individual values and mean ± S.D. number of subjects is in parentheses. * *p* < 0.05, ** *p* < 0.01, *** *p* < 0.001 by Mann–Whitney U-test.

In this sense, when the donors were divided into two groups depending on aortic Orai1 expression, the group with higher Orai1 expression (above median Orai1/β-actin ratio) was related to significantly higher SCr (Figure 7A–D) and lower GFR (Figure 7E–H) in respective kidney recipients, even at 24 months after transplantation. Conversely, recipients reaching SCr levels below 1.2 mg/dL at 12 months after transplantation received their kidneys from donors with significantly lower aortic expression of Orai1 (Figure 7I).

## 4. Discussion

The prevalence of renal diseases continues to rise due to population aging and the higher incidence of obesity, hypertension and diabetes [1,32]. In particular, CKD is a very common condition that affects millions of people in the world [1,2]. Kidney transplantation stands out as the definitive treatment option, offering clear superiority in terms of both survival and quality of life [3,4]. The increasing recipient demand and the limited supply of donors highlights the importance of revising strategies to optimize kidney transplantation to ensure the success of the grafts [33]. Renal transplant recipients who resume dialysis following graft failure have a shorter life expectancy than those who remain independent from dialysis [34]. In addition, 20% of patients waiting for a kidney are those whose first transplant failed [35]. Thus, studying factors that predict the success/failure of renal grafts seems even more important.

Among the multiple factors compromising kidney transplantation success [36,37,38], the most critical determinant is the quality of the organ itself [39]. Existing evidence suggests that donor age has a significant negative effect on transplant outcomes [37,38], although good long-term survival of kidney grafts from donors older than 60 years has been reported [40]. In this sense, alternative predictors beyond chronological age are needed [41].

It is well known that kidney function is closely related to vascular health and that CKD risk increases when CVRFs are present [12,32], but no studies have evaluated the relationship between the donor’s vascular function and the evolution of the grafts. In this study, we found that donors’ vascular function in very different vascular beds was significantly associated with SCr and GFR up to 12 months after transplantation. Thus, the present study demonstrates for the first time that systemic extrarenal vascular function of a kidney donor is a determinant for the recipient’s renal function in the short to mid-term. This concept is reinforced by the fact that donors situated in the best vascular function quartiles are related to better renal function in their respective graft recipients and that, conversely, kidney recipients achieving normal serum creatinine were related to donors with significantly better endothelial vasodilation and lower aortic contractility. This evidence supports the idea that a good donor vascular function could be predictive of the functional success of the transplanted kidney.

Specific vascular beds display significant heterogeneity in vulnerability and manifestations of the impact of CVRFs [11,29] as well as in the mechanisms of endothelial vasodilation. In this sense, we demonstrate an association of kidney recipient renal function with endothelial vasodilation in mesenteric small vessels where endothelium-derived hyperpolarization (EDH) is key for endothelial vasodilation [42] as well as in the corpus cavernosum, with almost complete dependence on NO [43]. The evaluation of cavernosal function is also of interest because it is a functional determinant of ED, which, in turn, is highly prevalent in renal failure and has been proposed as a sentinel marker of future CVD and general health [33].

However, in addition to the involvement of endothelial function in CVD, vasoconstriction plays an important role in clinical manifestations of vascular dysfunction [44]. Thus, the association of hypercontractility of donor aortae with poorer renal function in kidney recipients makes sense in the context of the relevance of the balance contractile/relaxant tone in vascular function.

To find possible confounders, we fitted these correlations to other possible covariables, finding that some of them exert significant influence as confounding variables. However, all the correlations of donors’ vascular function with renal parameters in recipients remained significant once the model was adjusted for all those variables, suggesting that vascular function provides independent predictive information on graft success. The adjusted model revealed that the association of donor vascular function and recipient renal function is independent of the evolution time. However, its significance weakens at 2-year evolution. This could be obviously due to the smaller sample size (lost to follow-up) but it should be considered that influences of the recipient’s environment and inherent conditions probably prevail on the longer term.

Considering the great influence of a donor’s vascular function on renal outcomes in a kidney recipient, markers of vascular function in the donor could serve as clues for predicting the result of the transplantation in terms of renal function in the recipient. In this sense, ADMA is considered a biomarker of endothelial dysfunction and CVD risk [13,14,15]. Indeed, we confirmed the association of plasma concentrations of ADMA with a significant decline in endothelial vasodilatory capacity of mesenteric microvessels of donors. This negative association was also obtained with TNF-α, which is also related to the development of vascular alterations and CVD [16,17]. Plasmatic concentrations of the calcium channel forming subunit, Orai1, also displayed a negative association with donor vasodilatory function. However, in contrast to ADMA and TNF-α, Orai1 was also related to an increase in donor aortic contractility. Upregulation of Orai proteins has been shown to contribute to vascular impairment related to aging in animal and human vessels [20,21] as well as to penile vascular alterations in erectile dysfunction and diabetes [18,19]. This is consistent with the associations of plasma concentrations of Orai1 with poorer vascular function in donors. Furthermore, despite the reported relationship of ADMA concentrations with worse renal function in elderly people [45] and poor prognosis in chronic kidney disease [46] as well as the increase in TNF-α in patients with diabetic nephropathy [47], Orai1 was the only donor biomarker able to predict worse renal function in the kidney recipients up to 6 months after transplantation. Interestingly, neither Orai1, ADMA nor TNF-α plasma concentration in kidney recipients influenced their SCr or GFR values. This fact highlights the relevance of donor phenotype in predicting kidney transplantation success at short/mid-term and targeting the donor to search for biomarkers helping to delineate such prediction. In this sense, the potential utility of donor Orai1 as a biomarker of the renal outcome in the kidney recipient is proposed.

In order to clarify the relationship of Orai1 with donor vascular function and kidney recipient renal function, we determined the expression of the Orai1 protein in aortic tissue from donors. Orai1 expression in aortic vascular tissue was related to increasing aortic vasoconstriction as well as inversely related to donor vasodilatory capacity determined in a different vascular bed, confirming vascular expression of Orai1 as a marker of systemic vascular function. Furthermore, aortic expression of Orai1 in donors was significantly associated with renal outcome in kidney recipients, predicting higher SCr and lower GFR even beyond the 12-month period following transplantation. This reinforces the concept of Orai1 as key for a donor’s vascular phenotype and as a marker of kidney function after transplantation.

The determinant role of a donor’s vascular phenotype (both vascular function and Orai1 plasma and vascular content) weakens at the longest observation period, probably indicating the gaining role of environmental or specific disease conditions in the recipient that would mask the relevance of the molecular signature of the kidney donor. On the other hand, the predictive accuracy could be influenced by the incidence of post-transplant viral infections, which are more frequent with the administration of immunosuppressants, among other risk factors [48].

A limitation of the present study was the impossibility to perform all determinations in all donors because not all types of samples could be obtained, always prevailing the medical procedures over the sample procurement for research. This resulted in a relatively low number of determinations for some analyses, especially at the longest observation period, when we have to acknowledge an important loss in follow-up. In addition, the design of the study does not allow for establishing a causative role of Orai1 on renal outcomes.

## 5. Conclusions

The present study provides insight into donor vascular phenotypes for determining kidney function after transplantation and points to Orai1 as a contributor to reduced vascular performance in kidney donors and as a marker of worse performance of transplanted kidneys in recipients. This could help to improve prognostic information on kidney transplantation. Further research to delineate the role of Orai1 in renal outcomes is warranted.

## Figures and Tables

**Table 1:** Main characteristics of subjects included in the study.

	Kidney Donors	Kidney Recipients
n	60	64
Age (yrs ± SD)	53.5 ± 16.1	59.1 ± 12.8
BMI (kg/m^2^ ± SD)	27.6 ± 5.3	26.2 ± 5.5
Female (%)	15 (25.0)	21 (32.8)
Diabetes (%)	9 (15.0)	19 (29.7)
Hypertension (%)	23 (38.3)	58 (90.6)
Dyslipidemia (%)	14 (23.3)	22 (34.4)
Peripheral vascular disease (%)	6 (10.0)	7 (10.9)
Coronary artery disease (%)	10 (16.7)	8 (12.5)
*Cause of kidney disease*		
Glomerulonephritis (%)		15 (23.4)
Diabetic nephropathy (%)		12 (18.8)
Polycystic kidney disease (%)		10 (15.6)
Nephroangiosclerosis (%)		9 (14.1)
CTIN (%)		9 (14.1)
FSGS (%)		5 (7.8)
Vasculitis (%)		2 (3.1)
Unknown (%)		2 (3.1)

BMI, body mass index; CTIN, chronic tubulointerstitial nephritis; FSGS, focal and segmental glomerulosclerosis; SD, standard deviation; n indicates number of subjects.

**Table 2:** Mixed regression model for evaluating the influence of donor co-variables on the correlations of donors’ vascular function with kidney function in recipients.

	HMA pEC_50_ BK		HA E_max_ NE		HCC pEC_50_ ACh	
***Serum Creatinine (mg/dL)***						
Time since transplant (months)	*p = 0.6977*	***p* = 0.0001**	*p = 0.8758*	***p* = 0.0017**	*p = 0.9800*	***p* = 0.0001**
Donor’s age (years)	***p < 0.0001***	***p* = 0.0094**	***p = 0.0058***	***p* = 0.047**	*p = 0.8442*	***p* = 0.0022**
Donor’s gender (male/female)	*p = 0.0842*	***p* = 0.0013**	***p < 0.0001***	***p* = 0.0187**		
Type of death (brain death/asystole)	***p = 0.0014***	***p* = 0.0006**	***p < 0.0001***	***p* = 0.0028**	*p = 0.0629*	***p* = 0.0011**
Donor SCr (mg/dL)	***p < 0.0001***	***p* = 0.0025**	***p = 0.0077***	***p* = 0.0055**	***p = 0.0221***	***p* = 0.0001**
HLA incompatibilities	*p = 0.6857*	***p* = 0.0002**	*p = 0.1737*	***p* = 0.0010**	*p = 0.1800*	***p* < 0.0001**
***GFR (mL/min/1.73 m^2^)***						
Time since transplant (months)	*p = 0.7490*	***p* < 0.0001**	*p = 0.9117*	***p* < 0.0001**	*p = 0.9700*	***p* < 0.0001**
Donor’s age (years)	***p = 0.0003***	***p* = 0.0007**	***p = 0.0500***	***p* = 0.0089**	*p = 0.9600*	***p* = 0.0011**
Donor’s gender (male/female)	*p = 0.1065*	***p* < 0.0001**	***p < 0.0001***	***p* = 0.0002**		
Type of death (brain death/asystole)	***p = 0.0016***	***p* < 0.0001**	***p < 0.0001***	***p* < 0.0001**	***p = 0.0151***	***p* = 0.0001**
Donor SCr (mg/dL)	***p < 0.0001***	***p* < 0.0001**	***p = 0.0045***	***p* = 0.0002**	***p = 0.0092***	***p* < 0.0001**
HLA incompatibilities	*p = 0.5564*	***p* <0.0001**	*p = 0.3196*	***p* < 0.0001**	*p = 0.0700*	***p* < 0.0001**

ACh: acetylcholine; BK: bradykinin; GFR: glomerular filtration rate, HA: human aorta; HCC: human corpus cavernosum; HMAs: human mesenteric arteries; NE: norepinephrine; SCr: serum creatinine; pEC_50_ indicates negative logarithm of molar concentration of BK or ACh (endothelium-dependent relaxation) causing half-maximal relaxation. E_max_ indicates maximal adrenergic contraction to NE. *p*-values of the different possible confounding variables are shown in italics (also in bold when significant): values less than 0.05 indicate that these variables behave as confusing for the initial correlations (i.e., covariables that influence association of donor vascular function with recipient renal function). The other *p*-values refer to the correlations of donor vascular function parameters with SCr and GFR in kidney recipients adjusted for each possible confounding variable. In all cases, these correlations remain significant (*p* < 0.05).

## Data Availability

The datasets generated and analyzed during the current study are not publicly available due to confidentiality but are available from the corresponding author on reasonable request.

## Supplementary Materials

The following supporting information can be downloaded at: https://www.mdpi.com/article/10.3390/cells14131005/s1, Figure S1: Stratification of kidney recipients based on endothelial vasodilation and adrenergic contractility of their respective donors confirms the determinant role of donor vascular function for predicting glomerular filtration rate (GFR) in recipients; Figure S2: Donor’s plasmatic concentrations of Orai1 but not those of ADMA or TNF-α are negatively associated with serum creatinine concentrations in kidney recipients; Figure S3: Concentrations of ADMA, TNF-α and Orai1 in plasma from kidney recipients failed to predict their serum creatinine concentrations after transplantation; Figure S4: Complete images of immunoblots for Orai1 and β-actin in donor aorta.

## Abbreviations

The following abbreviations are used in this manuscript:

ACh	Acetylcholine;
ADMA	Asymmetric dimethyl arginine;
BK	Bradykinin;
CKD	Chronic Kidney disease;
CTIN	Chronic tubulointerstitial nephritis;
CVD	Cardiovascular disease;
CVRF	Cardiovascular risk factor;
DAPI	Diamidino-2-phenylindole;
ECDs	Expanded criteria donors;
ED	Erectile dysfunction;
EDH	Endothelium-dependent hyperpolarization;
FSGS	Focal and segmental glomerulosclerosis;
GFR	Glomerular filtration rate;
HA	Human aorta;
HCC	Human corpus cavernosum;
HLA	Human leukocyte antigen;
HMA	Human mesenteric artery;
KHS	Krebs–Henseleit solution;
NE	Norepinephrine;
NO	Nitric oxide;
PE	Phenylephrine;
QoL	Quality of life;
SCr	Serum creatinine;
SD	Standard deviation;
SOCE	Store-operated calcium entry;
TNF-α	Tumor necrosis factor-α.
