# Supplementary material for: Orai1 Expression and Vascular Function in Kidney Donors Determine Graft Outcomes at Short/Mid-Term
Source: Cells. 2025 Jul 1;14(13):1005. doi: 10.3390/cells14131005 (PMC12249094; doi:10.3390/cells14131005)
Supplement: Supplementary file 1 [file cells-14-01005-s001.zip › cells-3675327-supplementary.pptx]

## Slide 1
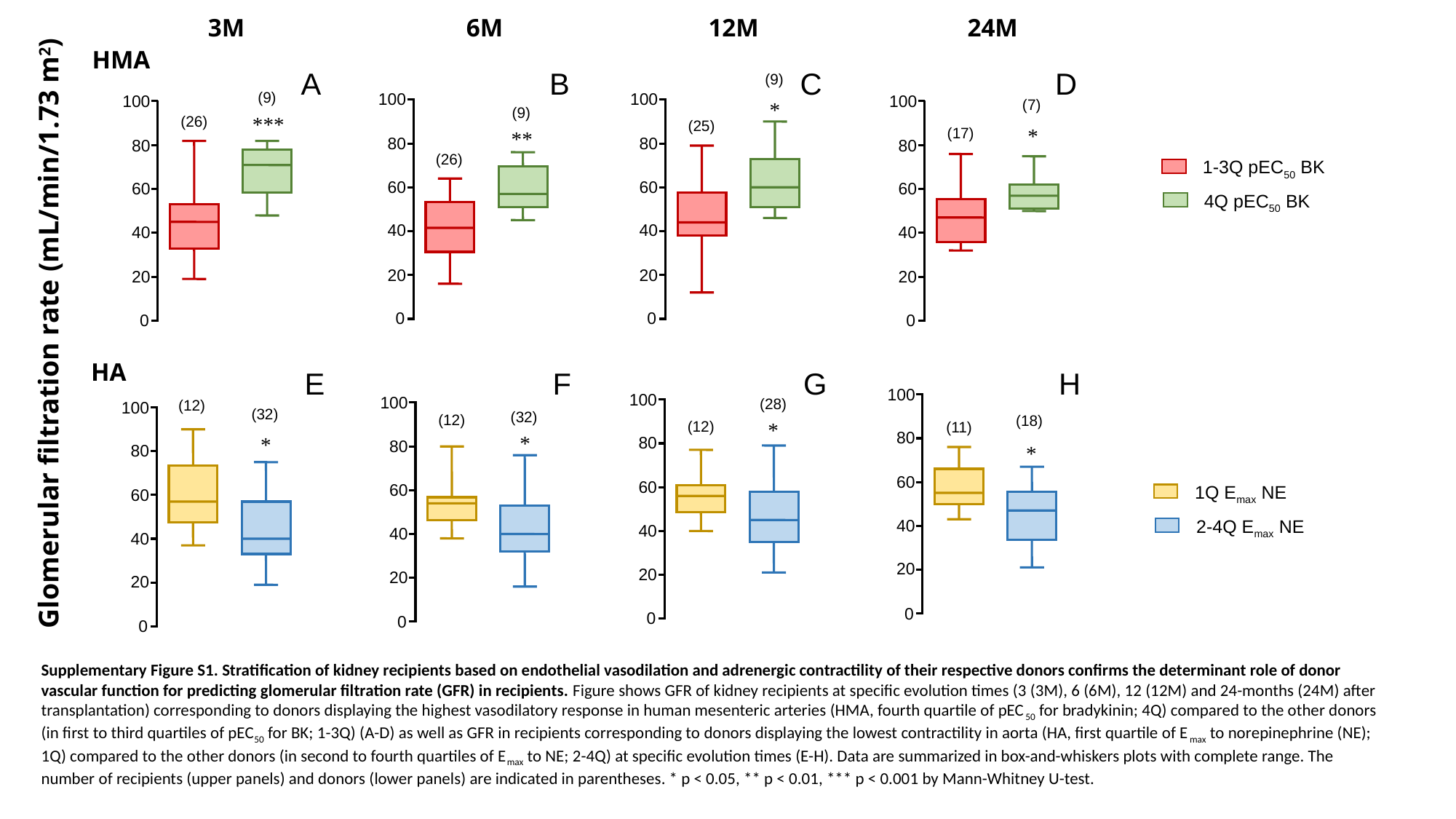

3M
6M
12M
24M
HMA
A
B
C
D
(9)
(9)
100
100
(7)
100
100
*
(9)
***
(26)
(25)
*
(17)
**
80
80
80
80
(26)
1-3Q pEC50 BK
60
60
60
60
4Q pEC50 BK
40
40
40
40
20
20
20
20
0
0
0
0
Glomerular filtration rate (mL/min/1.73 m2)
HA
E
F
G
H
100
100
(28)
(12)
100
100
(32)
(32)
(12)
(18)
(12)
*
(11)
*
*
80
80
*
80
80
60
60
60
1Q Emax NE
60
2-4Q Emax NE
40
40
40
40
20
20
20
20
0
0
0
0
Supplementary Figure S1. Stratification of kidney recipients based on endothelial vasodilation and adrenergic contractility of their respective donors confirms the determinant role of donor vascular function for predicting glomerular filtration rate (GFR) in recipients. Figure shows GFR of kidney recipients at specific evolution times (3 (3M), 6 (6M), 12 (12M) and 24-months (24M) after transplantation) corresponding to donors displaying the highest vasodilatory response in human mesenteric arteries (HMA, fourth quartile of pEC50 for bradykinin; 4Q) compared to the other donors (in first to third quartiles of pEC50 for BK; 1-3Q) (A-D) as well as GFR in recipients corresponding to donors displaying the lowest contractility in aorta (HA, first quartile of Emax to norepinephrine (NE); 1Q) compared to the other donors (in second to fourth quartiles of Emax to NE; 2-4Q) at specific evolution times (E-H). Data are summarized in box-and-whiskers plots with complete range. The number of recipients (upper panels) and donors (lower panels) are indicated in parentheses. * p < 0.05, ** p < 0.01, *** p < 0.001 by Mann-Whitney U-test.

## Slide 2
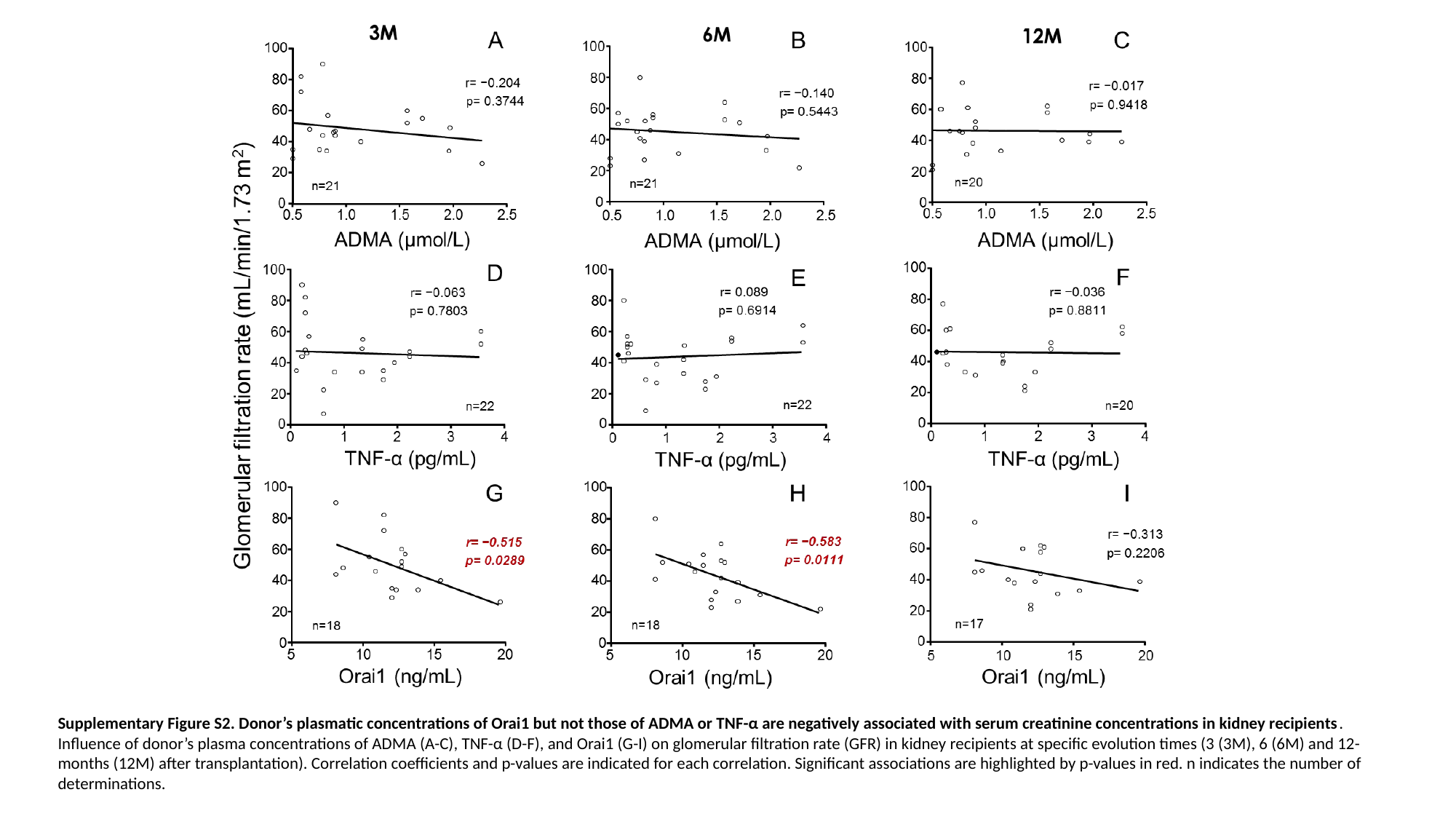

Supplementary Figure S2. Donor’s plasmatic concentrations of Orai1 but not those of ADMA or TNF-α are negatively associated with serum creatinine concentrations in kidney recipients. Influence of donor’s plasma concentrations of ADMA (A-C), TNF-α (D-F), and Orai1 (G-I) on glomerular filtration rate (GFR) in kidney recipients at specific evolution times (3 (3M), 6 (6M) and 12-months (12M) after transplantation). Correlation coefficients and p-values are indicated for each correlation. Significant associations are highlighted by p-values in red. n indicates the number of determinations.

## Slide 3
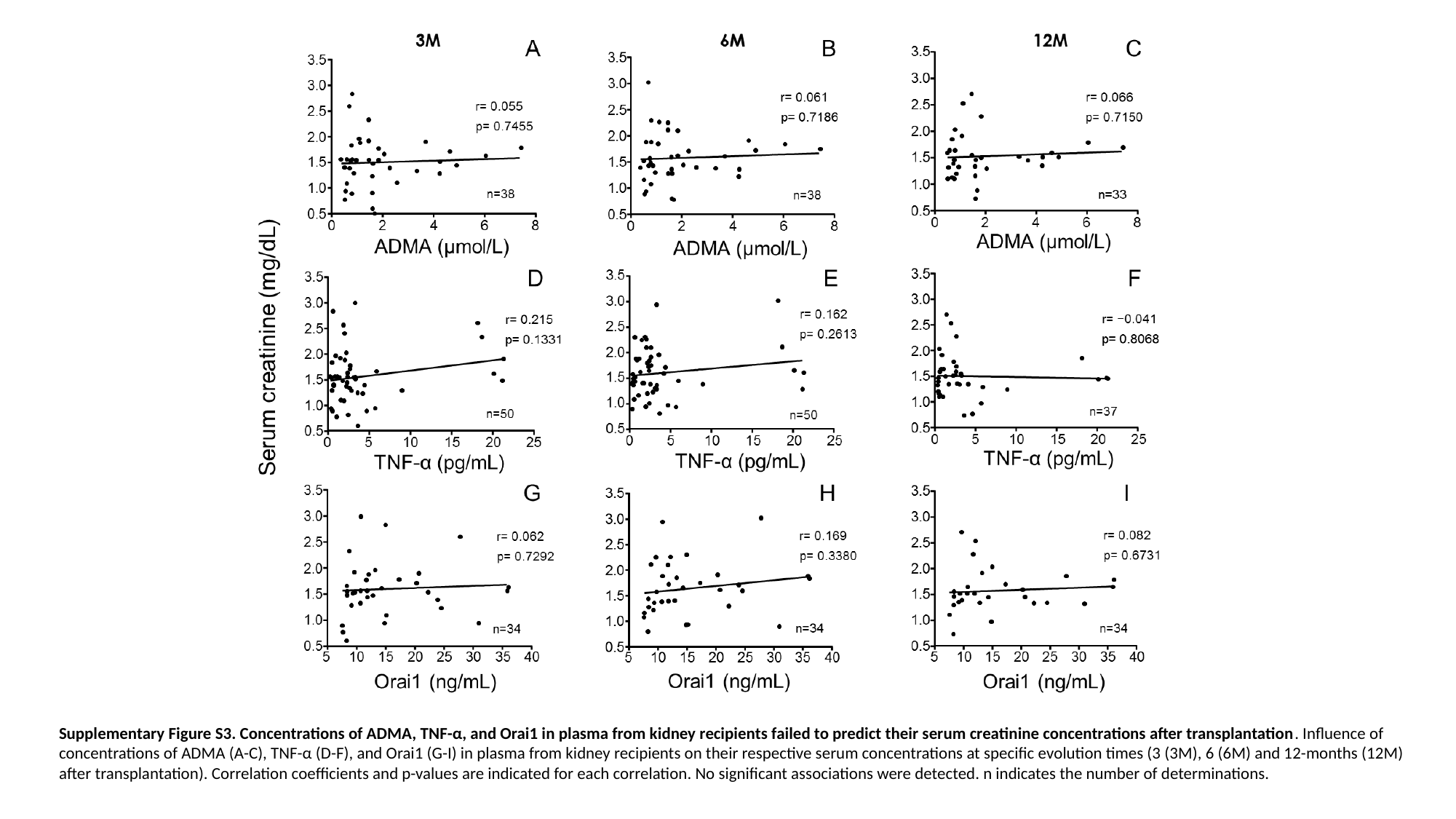

Supplementary Figure S3. Concentrations of ADMA, TNF-α, and Orai1 in plasma from kidney recipients failed to predict their serum creatinine concentrations after transplantation. Influence of concentrations of ADMA (A-C), TNF-α (D-F), and Orai1 (G-I) in plasma from kidney recipients on their respective serum concentrations at specific evolution times (3 (3M), 6 (6M) and 12-months (12M) after transplantation). Correlation coefficients and p-values are indicated for each correlation. No significant associations were detected. n indicates the number of determinations.

## Slide 4
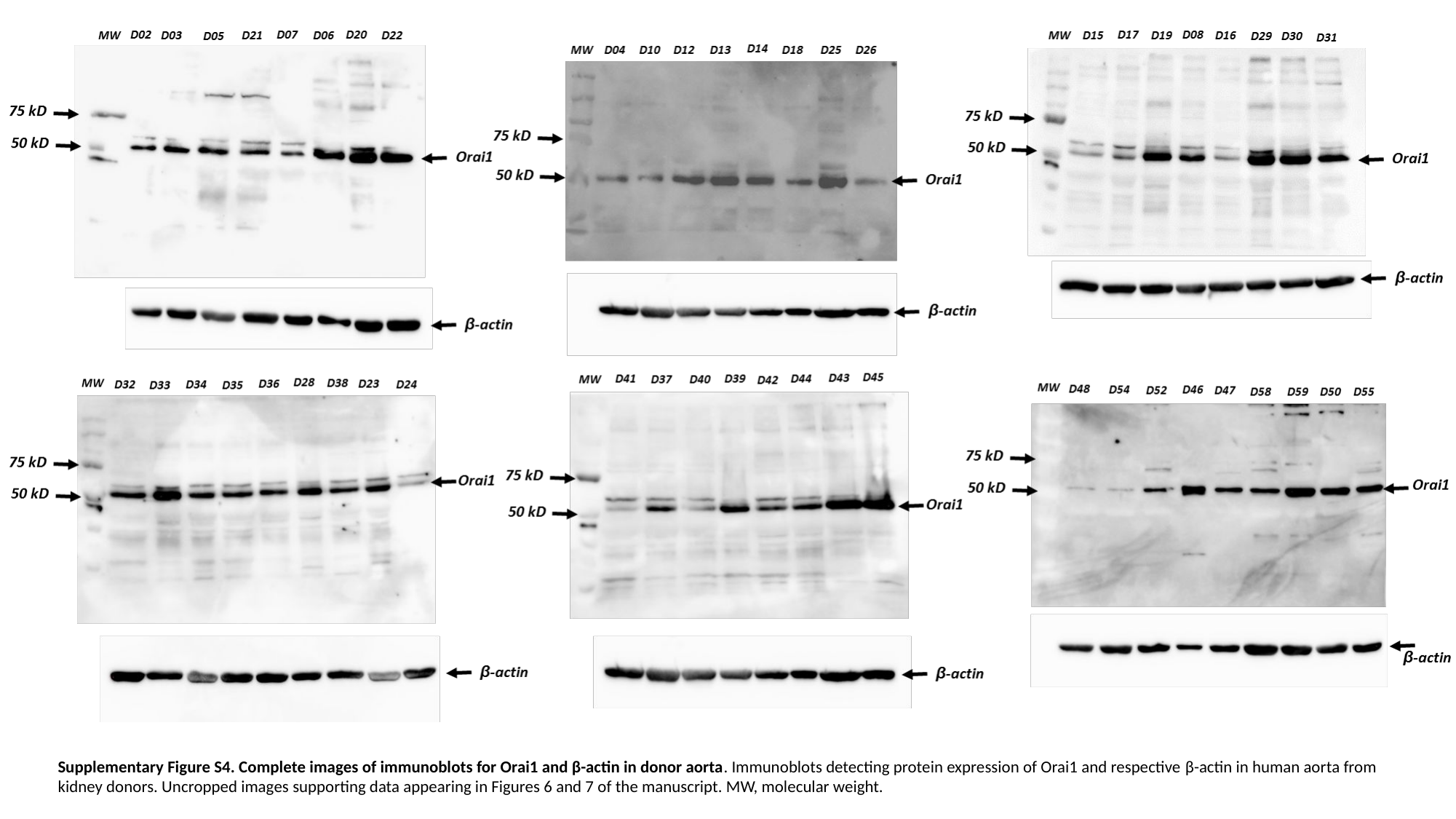

Supplementary Figure S4. Complete images of immunoblots for Orai1 and β-actin in donor aorta. Immunoblots detecting protein expression of Orai1 and respective β-actin in human aorta from kidney donors. Uncropped images supporting data appearing in Figures 6 and 7 of the manuscript. MW, molecular weight.
